# Glycerol contained in vaping liquids affects the liver and aspects of energy homeostasis in a sex‐dependent manner

**DOI:** 10.14814/phy2.15146

**Published:** 2022-01-25

**Authors:** Ariane Lechasseur, Mathilde Mouchiroud, Félix Tremblay, Gabrielle Bouffard, Nadia Milad, Marie Pineault, Michaël Maranda‐Robitaille, Joanie Routhier, Marie‐Josée Beaulieu, Sophie Aubin, Mathieu Laplante, Mathieu C. Morissette

**Affiliations:** ^1^ Quebec Heart and Lung Institute Université Laval Quebec Quebec Canada; ^2^ Faculty of Medicine Université Laval Quebec Quebec Canada; ^3^ Department of Medicine Université Laval Quebec Quebec Canada

**Keywords:** glycerol, liver, vaping

## Abstract

Vaping is increasingly popular among the young and adult population. Vaping liquids contained in electronic cigarettes (e‐cigarettes) are mainly composed of propylene glycol and glycerol, to which nicotine and flavors are added. Among several biological processes, glycerol is a metabolic substrate used for lipid synthesis in fed state as well as glucose synthesis in fasting state. We aimed to investigate the effects of glycerol e‐cigarette aerosol exposure on the aspects of glycerol and glucose homeostasis. Adult and young male and female mice were exposed to e‐cigarette aerosols with glycerol as vaping liquid using an established whole‐body exposure system. Mice were exposed acutely (single 2‐h exposure) or chronically (2 h/day, 5 days/week for 9 weeks). Circulating glycerol and glucose levels were assessed and glycerol as well as glucose tolerance tests were performed. The liver was also investigated to assess changes in the histology, lipid content, inflammation, and stress markers. Lung functions were also assessed as well as hepatic mRNA expression of genes controlling the circadian rhythm. Acute exposure to glycerol aerosols generated by an e‐cigarette increased circulating glycerol levels in female mice. Increased hepatic triglyceride and phosphatidylcholine concentrations were observed in female mice with no increase in circulating alanine aminotransferase or evidence of inflammation, fibrosis, or endoplasmic reticulum stress. Chronic exposure to glycerol e‐cigarette aerosols mildly impacted glucose tolerance test in young female and male mice. Fasting glycerol, glucose, and insulin remained unchanged. Increased pulmonary resistance was observed in young male mice. Taken together, this study shows that the glycerol contained in vaping liquids can affect the liver as well as the aspects of glucose and glycerol homeostasis. Additional work is required to translate these observations to humans and determine the biological and potential pathological impacts of these findings.

## Graphical abstract

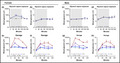

## 1 INTRODUCTION

Electronic cigarette (e‐cigarette) use, also known as vaping, is now a widespread habit about which we know very little of the safety and biological effects. In North America, e‐cigarette use has surpassed tobacco cigarette smoking among adolescents and young adults (Azagba, 2018; Azagba et al., 2019; East et al., 2018). Cases of severe lung injury, called “Electronic cigarette or vaping product use‐associated lung injury” or EVALI, suggest asymptomatic as well as symptomatic biological effects of vaping are to be expected. Therefore, as vaping is now common across many demographic groups, the need to identify the biological effects of this habit and the specific role of each constituent is crucial.

The major constituents of vaping liquids are propylene glycol, glycerol, nicotine, and a wide variety of flavoring chemicals. This liquid is aerosolized upon contact with a battery‐powered heat‐generating atomizer and inhaled by the user. The biological effects of nicotine are well established, thanks to decades of research to tobacco smoking, and flavoring agents greatly vary from a vaping liquid to another (Tierney et al., 2016). Propylene glycol and/or glycerol are the common constituents of every vaping liquid, which act as vehicles for nicotine and flavors and also facilitate aerosolization.

Main focus has been placed on investigating pulmonary effects of e‐cigarette emissions, since its aerosols are inhaled. However, a study by our research group showed that both glycerol and propylene glycol have the ability to change the expression of genes controlling the circadian rhythm in the lungs, but also in the liver, kidneys, and skeletal muscles, suggesting systemic effects independent of nicotine or flavors (Lechasseur et al., 2017).

While propylene glycol is a man‐made chemical, glycerol is found in large quantities in living organisms and is involved in numerous metabolic processes. Among others, glycerol acts as a substrate for gluconeogenesis during fasting periods to support glucose synthesis in the liver and maintain glycemia. It also acts as a building block for several lipid species including triglycerides and phospholipids (reviewed in: [Rui, 2014]). To date, only a few studies investigated the impact of glycerol vaping on energy metabolism, and none included both male and female mice of various ages.

In this study, we aimed at investigating in male and female mice the impact of glycerol vaping on the liver and aspects of energy homeostasis in a well‐established model of e‐cigarette exposure. We found that glycerol can accumulate in the blood of female mice exposed to glycerol aerosols generated by an e‐cigarette, a phenomenon not observed in males. We also found that long‐term exposure increases hepatic triglyceride and phosphatidylcholine contents in younger and older female mice, but not in their male counterparts. We also found mild alterations in glucose and glycerol tolerance tests, also showing sexual dimorphism. Interestingly, these metabolic effects were happening in the absence of major changes in lung functions, suggesting metabolic effects of glycerol vaping can precede its effects on lung physiology. Finally, this study suggests that glycerol vaping can impact liver and energy metabolism, especially in females.

## 2 METHODS

### 2.1 Glycerol e‐cigarette aerosol exposure

Male and female young (Bass & Takahashi, 2010) and adult (Folch et al., 1957) C57bl/6N mice were purchased from Charles River (St‐Constant, PQ, Canada). Mice were housed in 12:12 light/dark cycles (light periods from 6 am to 6 pm) with access to food (Teklad Global 18% Protein Rodent Diet; #2018) and water ad libitum. Mice were housed according to the Canadian Council for Animal Care (CCAC) guidelines and Université Laval's Animal Research Ethics Board approved all procedures (Animal utilization protocol #2014121‐2).

Exposure to e‐cigarette aerosols took place using a whole‐body exposure system as previously described (Lechasseur et al., 2017, 2020). A pump and pinch valve are controlled by a programmable automated system (InExpose control board; SCIREQ Scientific Respiratory Equipment Inc) to take two 70 ml puffs per minute from a commercial e‐cigarette. The puffs are then mixed with room air (bias flow of 3 L/min) and sent in the whole‐body exposure chamber by laminar flow where mice freely breathe the aerosols. E‐cigarette device used was a draw‐activated UWELL by Caliburn, with a refillable open pod cartridge. Coil resistance was of 1.4 Ω and battery power was of 11 W. USP grade 100% glycerol was used as e‐liquid, with no nicotine or flavoring added. Mice were exposed acutely (a single 2‐h exposure) or chronically for two consecutive hours between 13:00 and 15:00, 5 days a week, for 9 weeks.

### 2.2 Blood glycerol assessment following inhalation and gavage

Mice were fasted for 12 h (from 20:00 to 08:00) prior to experiments. Mice (*n* = 3–4/group) were then subjected to a 2‐h glycerol e‐cigarette aerosol exposure and blood was drawn before, at mid‐exposure, after the exposure, and 30 as well as 60 min following the end of the exposure. Other groups also received glycerol by gavage (2, 0.7, 0.2, 0.07, or 0 g/kg of glycerol in water). Blood was collected from the tail vein before and at 30, 60, 90, and 120 min following the gavage. Glucose was measured using a glucometer (Accu‐Chek Performa; Roche).

### 2.3 Glycerol and glucose tolerance tests

To assess the impact of glycerol aerosol exposure on glucose and glycerol circulating levels, glucose and glycerol tolerance tests were conducted following 6 and 7 weeks of exposure, respectively. Mice were fasted for 12 h (from 20:00 to 08:00) prior to experiments. For glycerol tolerance test, all mice were subjected to a 2 g/kg glycerol intraperitoneal injection. Glucose was measured using a glucometer (Accu‐Chek Performa; Roche) and blood was collected from the tail vein before and at 30, 60, 90, and 120‐min post‐injection. For glucose tolerance test, all mice were injected with 1 g/kg of d‐glucose. Glucose was measured using whole blood and a glucometer (Accu‐Chek Performa; Roche) before and at 15, 30, 45, 60, 90, and 120‐min post‐injection. Serum was isolated from blood and treated with Carrez Clarification Reagent Kit (ab202373; Abcam). Free glycerol was measured according to the manufacturer's instructions (ab65337; Abcam).

### 2.4 Lung function measurement

Mice were fasted at 20:00 on the day of the last exposure (5 h after the end of exposure). Starting at 08:00 the next day (17 h after the last exposure), mice were weighted, and glucose was measured using a glucometer (Accu‐Chek Performa; Roche). Mice were then anesthetized with 100 mg/kg ketamine and 10 mg/kg xylazine. Lung function parameters were attested by FlexiVent (Scireq). Mice were tracheotomized with an 18‐gage blunted needle, mechanically ventilated at a respiratory rate of 150 breaths/min and a tidal volume of 10 ml/kg, with a pressure limit of 30 cmH_2_O. Muscle paralysis was achieved using pancuronium (2 mg/kg; Sandoz) to prevent respiratory efforts during the measurement. The following sequence of measures was repeated three times: Deep inflation, Snapshot‐150, Quick Prime‐3, and Pressure/Volume loop to obtain lung resistance, compliance and elastance, Newtonian resistance, tissue resistance, tissue elastance, a pressure–volume curve, inspiratory capacity, and hysteresis.

### 2.5 Sample harvesting and processing and histology assessment

In anesthetized mice, blood was collected from the retro‐orbital vein to obtain serum (incubated at 37°C for 60 min then spun for 10 min at 12,000 *g*). Mice were then euthanized at random by exsanguination by severing the descending aorta. Liver and adipose tissue (ovarian/epididymal, inguinal, and retroperitoneal) were harvested, weighted, and snap frozen for further analysis. A portion of the liver was placed in 10% formalin for 3 days prior transfer to 70% ethanol and paraffin‐embedding. Liver sections were stained with hematoxylin and eosin (H&E).

### 2.6 Triglyceride, phosphatidylcholine, insulin, and ALT measurements

Liver lipids were extracted from tissues as described by Folch et al. (1957) and resuspended in isopropanol. Hepatic triglyceride levels were determined with a standard assay kit (TR22421; Thermo Fisher Scientific) according to the manufacturer's instructions. Hepatic phosphatidylcholine levels were measured with a standard assay kit (STA‐600; Cell Biolabs) according to the manufacturer's instructions. Fasting insulin was assessed (Ultra Sensitive Mouse Insulin ELISA Kit; Crystal Chem). Blood alanine aminotransferase (ALT) activity was assessed according to the manufacturer's instructions (ALT Activity Assay, MAK052; Sigma‐Aldrich).

### 2.7 Quantitative PCR

Total RNA was extracted using TRIzol reagent (Fisher Scientific). RNA quantification and purity were assessed with the Synergy H1 plate reader and the Gen5 software (BioTek). RNA integrity was assessed by gel electrophoresis. One microgram of RNA was converted into cDNA using the iScript Advanced cDNA synthesis kit (Bio‐Rad). qPCR analyses were performed using SsoAdvanced Universal SYBR Green Supermix (Bio‐Rad) and primers (IDT) at 300 nM (see Table 1 for primer information). qPCRs were performed using a CFX384 Touch qPCR System (Bio‐Rad) as follows: 95°C for 3 min, followed by 40 cycles of 95°C for 10 s and 57–60°C for 30 s followed by a melt curve to assure specificity. For each gene, a temperature gradient was made to define the ideal annealing temperature. A calibration curve from pooled liver samples was also made to determine the PCR efficiency and a *r* ^2^. All qPCR efficiencies were between 90% and 110%, with *r* ^2^ values ranging between 0.97 and 1.00. Data were acquired and analyzed with the CFX Manager software (version 3.1). For each gene, *C*_q_ values were determined as the intercept of each amplification curve with the threshold established in the calibration curve. All reactions were performed in triplicate (SD < 0.3). Gene expression levels were assessed using *hprt* and *rplp0* reporter genes using the ∆∆*C*_q_ method.

**TABLE 1:** Primer sequences

Gene symbol	Sequence accession number	Amplicon size (pb)	Primer sequences	Annealing temperature (°C)
Acaca	NM_133360	93	For: AAC ATC CCC ACG CTA AAC AG	59
			Rev: GTC CAA CAG AAC ATC GCT GA	
Agpat9	NM_172715	148	For: ACC ATA ACA AGC AGT ACA GAC C	59
			Rev: GCT CTC TGA ATG ATC CCC ATC	
Atf6	NM_001081304	204	For: GAA CTT CGA GGC TGG GTT CA	60
			Rev: TCC AGG GGA GGC GTA ATA CA	
Aqp9	NM_022026	123	For: TCA CGG GAG AAA ATG GAA CG	59
			Rev: TGG CAA AGA CAA TCA GAA GGA	
Arntl	NM_007489	100	For: CGG TCA CAT CCT ACG ACA AAC	60
			Rev: CAG AAG CAA ACT ACA AGC CAA C	
Ccl2	NM_011333.3	142	For: AAC TAC AGC TTC TTT GGG ACA	57
			Rev: CAT CCA CGT GTT GGC TCA	
Cpt1a	NM_013495	119	For: CAG CAA GAT AGG CAT AAA CGC	59
			Rev: AGT GTC CAT CCT CTG AGT AGC	
Ctgf	NM_001901	140	For: TTG ACA GGC TTG GCG ATT	60
			Rev: GTT ACC AAT GAC AAT ACC TTC T	
Ddit3	NM_007837	176	For: TGC AGA TCC TCA TAC CAG GC	60
			Rev: CCA GAA TAA CAG CCG GAA CCT	
G6pc	NM_008061	108	For: GGA GGC TGG CAT TGT AGA TG	57
			Rev: TCT ACC TTG CTG CTC ACT TTC	
Gk	NM_008194	124	For: CCA ACG AAG TTT CAC TGC AC	57
			Rev: TGA CCT AAG AAC CCA GTC TAC T	
Hprt	NM_013556	125	For: AGC AGG TCA GCA AAG AAC T	57
			Rev: CCT CAT GGA CTG ATT ATG GAC A	
Ldlr	NM_001252659	132	For: TGC ATT TTC CGT CTC TAC ACT	57
			Rev: CAA CGC AGA AGC TAA GGA TGA	
Nr1d1	NM_145434	101	For: GAG CCA CTA GAG CCA ATG TAG	57
			Rev: CCA GTT TGA ATG ACC GCT TTC	
Nr1d2	NM_011584	112	For: ACA GTT CTC ATT CTT CAG GCA	57
			Rev: GGC ATC AGG ATT CCA CTA TGG	
Pck1	NM_011044	144	For: GCG AGT CTG TCA GTT CAA TAC C	57
			Rev: GGA TGT CGG AAG AGG ACT TTG	
Per1	NM_011065	133	For: CTT TGC TTT AGA TCG GCA GTG	57
			Rev: CTT CCT CAA CCG CTT CAG A	
Per2	NM_011066	118	For: TGA GGT AGA TAG CCC AGG AG	57
			Rev: GCT ATG AAG CGC CTA GAA TCC	
Per3	NM_011067	114	For: CTC TTC TCT CTG TCT CCA CCT	60
			Rev: TCC AAC TCA GCT TCC TTT CTG	
Rplp0	NM_007475	96	For: ATC ACA GAG CAG GCC CTG CA	57
			Rev: CAC CGA GGC AAC AGT TGG GT	
Tnf	NM_013693	145	For: AGA CCC TCA CAC TCA GAT CA	57
			Rev: TCT TTG AGA TCC ATG CCG TTG	

### 2.8 Statistical analysis

Two‐sided *t*‐tests were performed for two‐group comparisons. Regarding glucose and glycerol tolerance tests, two‐way ANOVA for independent variables “time” and “exposure type” was performed. If the “exposure type” variable was significant (*p* value <0.05), a Šídák's multiple comparison post hoc test was then performed for each timepoint. Statistically significant differences were considered if *p* < 0.05. All statistical analyses were performed using Prism 9 from GraphPad Software, Inc.

## 3 RESULTS

### 3.1 Glycerol e‐cigarette aerosol exposure impacts circulating glycerol levels

**FIGURE 1:**
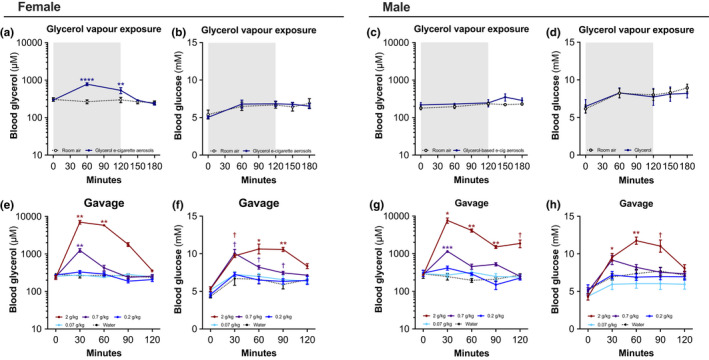
Impact of glycerol e‐cigarette aerosol inhalation and glycerol gavage on blood glycerol and glucose concentrations. Six‐week‐old female and male mice (*n* = 3–4) were administered glycerol in different forms. Mice were exposed for 2 h to glycerol e‐cigarette aerosols (blue circles) or room air (white open circles). Exposure period is represented by the shaded region. Blood glycerol (a, c) and blood glucose (b, d) concentrations were measured. Mice received a glycerol gavage containing 2 g/kg (red), 0.7 g/kg (purple), 0.2 g/kg (dark blue), 0.07 g/kg (light blue), or water (white open circles). Blood glycerol (e, g) and blood glucose (f, h) concentrations were measured. Data are presented as mean ± SEM. Two‐way ANOVA with Šídák's multiple comparison post test was performed comparing experimental groups to control group: *p < 0.05; **p < 0.01; ***p < 0.001. Two‐sided Student's *t*‐tests were performed for two‐group comparisons: ^†^ *p* < 0.05; ^†^ *p* < 0.01

We first sought to investigate the impact of an acute glycerol e‐cigarette aerosol exposure as well as glycerol gavage on circulating glycerol and glucose levels. Mice were fasted 12 h prior and blood was drawn at different timepoints to assess glucose and glycerol concentrations. Female mice exposed to glycerol e‐cigarette aerosols showed a significant increase in serum glycerol, peaking at the 60‐min mark and returning to baseline levels at 150, or 30 min following the end exposure (Figure 1a). Interestingly, no increase in blood glycerol was detected in male mice following exposure (Figure 1c). Blood glucose remained unchanged for female (Figure 1b) and male (Figure 1d) mice. Gavage of 2 g/kg of glycerol increased blood glycerol in female and male mice (Figure 1e,g) and subsequently increased blood glucose in both sexes (Figure 1f,h). While we cannot statistically compare these separate experiments, this shows that exposure to glycerol e‐cigarette aerosols differently affects female and male mice circulating glycerol levels while gavage leads to similar outcomes in both sexes.

### 3.2 Exposure to glycerol e‐cigarette aerosols does not affect body weight

**FIGURE 2:**
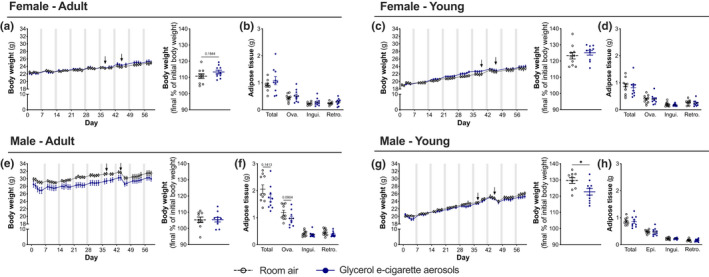
Glycerol e‐cigarette aerosol exposure does not change body weight. Twelve‐week‐old (adult) and 6‐week‐old (young) female and male mice (*n* = 9–10) were exposed to room air (white open circles) or glycerol e‐cigarette aerosols (blue circles) for 2 h a day, 5 days a week for 9 weeks. Mice were weighted every morning at the same time to ensure reproducibility (a, c, e, g). Upon euthanasia, ovarian (Ova)/epididymal (Epi), inguinal (Ingui), and retroperitoneal (Retro) adipose tissue were weighted for room air (black and gray boxes) and glycerol e‐cigarette aerosol‐exposed mice (blue boxes) (b, d, f, h). Arrows represent fasting period mice underwent for glycerol and glucose tolerance tests. Gray shaded regions represent weekends, where no exposure took place. Data are presented as mean ± SEM. Two‐way ANOVA with Šídák's multiple comparison post test was performed for body weight curves. Two‐sided Student's *t*‐tests were performed for two‐group comparisons. *p < 0.05

Studies have shown that age can change the response to diets, with mice being less susceptible to gaining weight when initiated early to a high‐fat diet (Dong et al., 2017; Garcia et al., 2016). As glycerol can be used as a source of energy, we investigated the impact of chronic 9‐week glycerol e‐cigarette aerosol exposure on weight gain, considering sex as well as age at exposure onset as variables. Overall, we found no changes in weight gain between controls and mice exposed to glycerol e‐cigarette aerosols (Figure 2a,c,e,g). In addition, glycerol e‐cigarette aerosol exposure did not affect adipose tissue mass in all four groups (Figure 2b,d,f,h).

### 3.3 Exposure to glycerol e‐cigarette aerosols increases hepatic triglycerides and phosphatidylcholine concentrations in female mice

**FIGURE 3:**
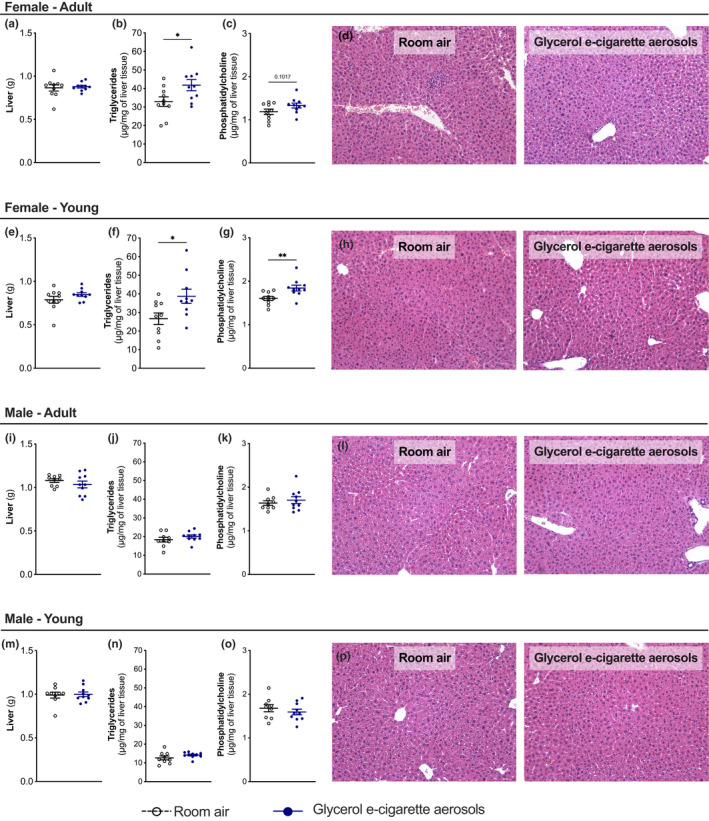
Glycerol e‐cigarette aerosol exposure increases hepatic triglyceride and phosphatidylcholine content in female mice. Twelve‐week‐old (adult) and 6‐week‐old (young) female and male mice (*n* = 9–10) were exposed to room air (white open circles) or glycerol e‐cigarette aerosols (blue circles) for 2 h a day, 5 days a week for 9 weeks. Upon euthanasia, liver weight was measured (a, e, i, m). Hepatic triglycerides (b, f, j, n) and phosphatidylcholine (c, g, k, o) levels were measured. Hematoxylin and eosin (H&E) staining of formalin‐fixed paraffin‐embedded liver sections was made (d, h, l, p). Data are presented as mean ± SEM. Two‐sided Student's *t*‐tests were performed for two‐group comparisons. *p < 0.05; **p < 0.01

Since the liver is a key organ in glycerol metabolism, livers were collected following the chronic 9‐week exposure to glycerol e‐cigarette aerosols. Liver weight remained similar between control and exposure groups, even when accounting for body weight (Figure 3a,e,i,m). Hepatic triglyceride concentrations were increased in glycerol e‐cigarette aerosol‐exposed young and adult female mice (Figure 3b,f), but not in male mice (Figure 3j,n). Similarly, hepatic phosphatidylcholine levels were increased in glycerol e‐cigarette aerosol‐exposed young and adult female mice (Figure 3c,g), but not in male mice (Figure 3k,o). This increased hepatic lipid accumulation in female mice was not associated with marked histologic changes (Figure 3d,h,l,p). Trying to identify potential transcriptional changes in key genes involved in lipid metabolism, we found no changes in liver mRNA levels for *ldlr*, involved in lipid transport, *agpat9*, involved in triglyceride synthesis, or *acaca*, involved in de novo lipogenesis. While there were no changes for females or for young males, adult male mice exposed to glycerol e‐cigarette aerosols showed increased liver mRNA for *cpt1a*, involved in mitochondrial β‐oxidation (Figure S1).

### 3.4 Exposure to glycerol e‐cigarette aerosols does not induce classical pathogenic inflammatory or stress markers in the liver

**FIGURE 4:**
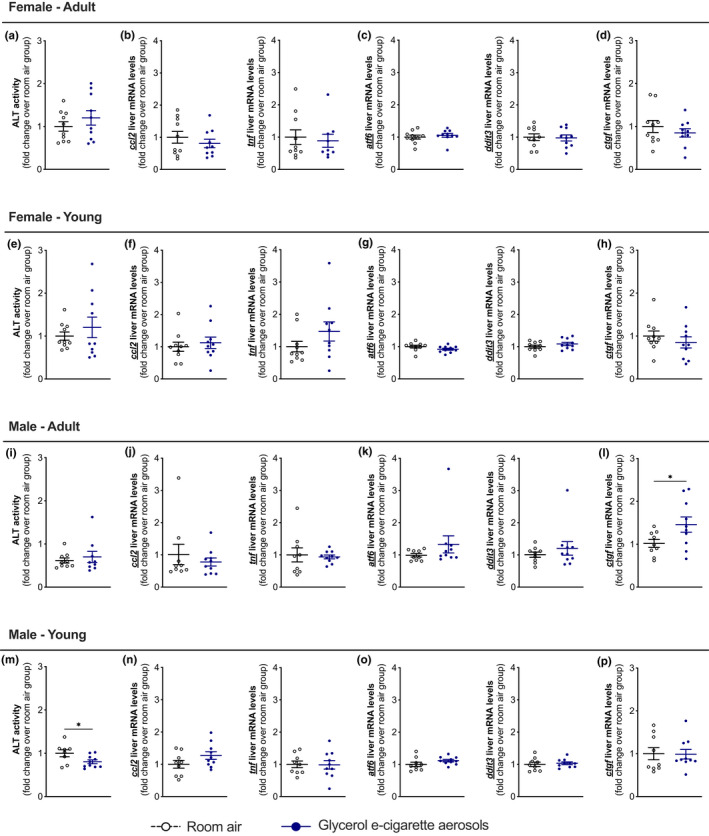
Impact of glycerol e‐cigarette aerosol exposure on liver inflammation, endoplasmic reticulum stress, and remodeling. Twelve‐week‐old (adult) and 6‐week‐old (young) female and male mice (*n* = 9–10) were exposed to room air (white open circles) or glycerol e‐cigarette aerosol (blue circles) for 2 h a day, 5 days a week for 9 weeks. Blood alanine aminotransferase activity (ALT) was measured (a, e, i, m). Hepatic expression level for inflammatory markers *ccl2* and *tnf* (b, f, j, n), endoplasmic reticulum stress markers *atf6* and *ddit3* (c, g, k, o), and remodeling marker *ctgf* was measured by qPCR analysis (b, e, h, k). Data are presented as mean ± SEM. Two‐sided Student's *t*‐tests were performed for two‐group comparisons. *p < 0.05

To determine if triglyceride accumulation induced by chronic exposure to glycerol e‐cigarette aerosols in the liver of female mice‐induced pathogenic processes, we assessed hepatic inflammatory markers as well as markers of endoplasmic reticulum stress known to be associated with hepatic steatosis. The activity for circulating ALT, a biomarker for liver damage, remained unchanged in female mice and adult male mice (Figure 4a,e,i), even slightly lower in young male mice (Figure 4m). Hepatic mRNA levels for the pro‐inflammatory mediators C‐C motif chemokine ligand 2 (*ccl2*) and tumor necrosis factor (*tnf*) were similar between control and exposed groups (Figure 4b,f,j,n). Hepatic mRNA levels for endoplasmic reticulum stress markers activating transcription factor 6 (*atf6*) and DNA damage‐inducible transcript 3 protein (*ddit3*) were also similar (Figure 4c,g,k,o). Hepatic mRNA levels for connective tissue growth factor (*ctfg*) were higher in adult males (Figure 4l) but not in other groups (Figure 4d,h,p).

### 3.5 Exposure to glycerol e‐cigarette aerosols does not change fasting glycerol and glucose metabolism

**FIGURE 5:**
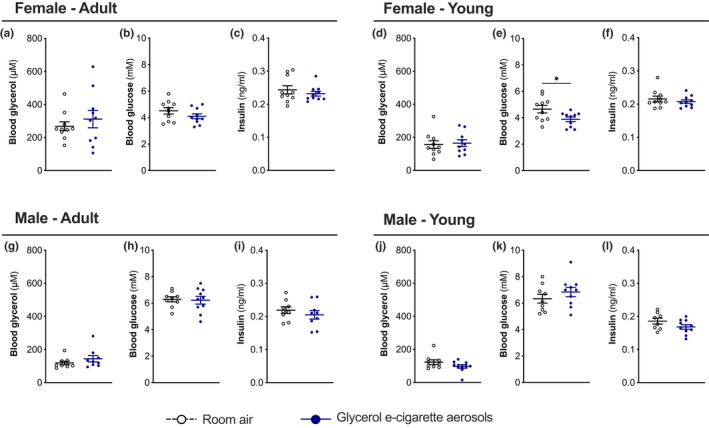
Impact of glycerol e‐cigarette aerosol exposure on fasting glycerol, glucose, and insulin concentrations. Twelve‐week‐old (adult) and 6‐week‐old (young) female and male mice (*n* = 9–10) were exposed to room air (white open circles) or glycerol e‐cigarette aerosol (blue circles) for 2 h a day, 5 days a week for 9 weeks. Upon euthanasia, fasting blood glycerol (a, d, g, j), glucose (b, e, h, k), and insulin (c, f, i, l) were assessed. Data are presented as mean ± SEM. Two‐sided Student's *t*‐tests were performed for two‐group comparisons. *p < 0.05

We then investigated if repeated exposure to inhaled aerosols of glycerol generated by an e‐cigarette could affect fasting glycerol and glucose circulating levels. Following 9 weeks of exposure, fasting blood glycerol levels remained similar between control and exposed groups (Figure 5a,d,g,j). Exposed young female mice showed slightly reduced fasting glucose levels compared to controls (Figure 5e), with similar fasting glucose between exposed and control adult female mice and male mice of both age groups (Figure 5b,h,k). Insulin concentrations were similar between all sex and age control and exposed groups (Figure 5c,f,i,l). Trying to identify potential transcriptional changes in key genes involved in glycerol uptake and metabolism, we found no variations in hepatic mRNA levels for *aqp9* and *gk* in adult and young female mice (Figure S1). However, exposed young and adult male mice showed a slight increase in *aqp9* liver mRNA levels compared to control groups. Both exposed adult and young male mice showed *gk* liver mRNA levels similar to the control groups (Figure S1). Hepatic mRNA levels for gluconeogenic genes *g6p6* and *pck1* remained similar between exposed and control mice for all four sex and age groups (Figure S1).

### 3.6 Exposure to glycerol e‐cigarette aerosols changes glycerol and glucose tolerance

**FIGURE 6:**
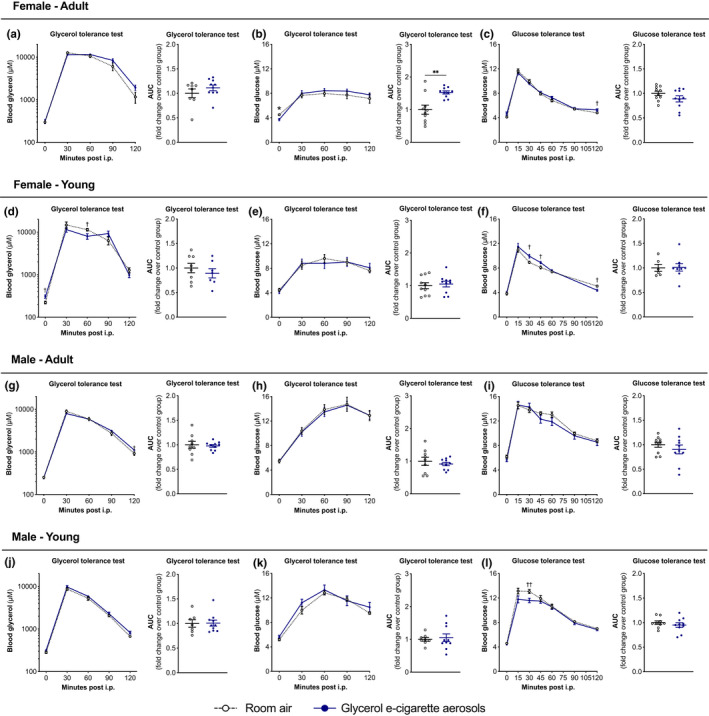
Impact of glycerol e‐cigarette aerosol exposure on glycerol and glucose tolerance. Twelve‐week‐old (adult) and 6‐week‐old (young) female and male mice (*n* = 9–10) were exposed to room air (white open circles) or glycerol e‐cigarette aerosol (blue circles) for 2 h a day, 5 days a week for 9 weeks. After 6 weeks of exposure, mice were injected with 2 g/kg of glycerol in a saline solution and blood glycerol (a, d, g, j) and blood glucose (b, e, h, k) were assessed. After 7 weeks of exposure, mice were injected with 1 g/kg of d‐glucose in a saline solution and blood glucose (c, f, i, l) was assessed. AUC = area under the curve. Data are presented as mean ± SEM. Two‐way ANOVA with Šídák's multiple comparison post test was performed for tolerance curves: *p < 0.05; **p < 0.01. Two‐sided Student's *t*‐tests were performed for two‐group comparisons at each timepoint and AUC data: ^†^ *p* < 0.05; ^††^ *p* < 0.01

We further assessed the impact of chronic exposure to glycerol e‐cigarette aerosols on glycerol metabolism. We first evaluated the ability of mice to process glycerol using a glycerol tolerance test. Based on a previously published study (Kosuga et al., 2011), all mice were fasted for 12 h after 6 weeks of glycerol e‐cigarette aerosol exposure. Mice were injected with 2 g/kg of glycerol and blood was drawn to assess blood glycerol and glucose levels. We found young female mice exposed to glycerol e‐cigarette aerosols metabolized injected glycerol differently than room air controls, with close to significant differences in adult female mice (Figure 6a,d). Adult male mice showed increased glycerol concentrations at the 120‐min mark, with similar trends in young male mice (Figure 6g,j). In all groups, changes found in glycerol concentration did not transpose into different glucose concentrations during the glycerol tolerance test (Figure 6b,e,h,k).

We next sought to investigate if glycerol e‐cigarette aerosol‐exposed mice had normal glucose tolerance. After 7 weeks of glycerol e‐cigarette aerosol exposure, mice were fasted for 12 h. Mice were injected with 1 g/kg of d‐glucose and blood glucose concentration was measured. Adult female and male did not show changes in glucose tolerance (Figure 6c,i). Young female mice showed increased blood glucose concentration in the first hour of the procedure, later to be decreased at the 120‐minute mark (Figure 6f). Young male mice showed decreased blood glucose concentration in the first hour of the procedure, later to return to similar levels as of room air‐exposed mice (Figure 6l).

### 3.7 Glycerol e‐cigarette aerosol exposure alters pulmonary functions in young male mice

To investigate how exposure to glycerol e‐cigarette aerosols was affecting the lungs, we assessed multiple parameters of lung functions in situ. While there were no changes between exposed and control female or adult male mice, young male mice exposed to glycerol e‐cigarette aerosols showed increased lung resistance and tissue damping, with decreased tissue damping (Figure S1a–d).

### 3.8 Exposure to glycerol e‐cigarette aerosols changes the expression of genes regulating the circadian rhythm in the liver of young male and female mice

Since we observed several impacts of sex and age on the liver and aspect of energy homeostasis, we wanted to revisit how the expression of genes regulating the circadian rhythm was affected in the liver. We found no differences between adult male and female mice (Figure S2a,c). Interestingly, we found that young mice exposed to glycerol e‐cigarette aerosols, both females and males, depicted changes in circadian gene expression, with changes for *arntl*, *per2*, and *per3* for females (Figure S2b) and *nr1d1* and *nr1d2* for males (Figure S2d).

## 4 DISCUSSION

In this study, we aimed to investigate the effects of e‐cigarette aerosols on the liver and aspects of energy homeostasis in non‐pathological conditions using mice. More specifically, as glycerol is a direct substrate for several metabolic processes, we assessed the effects of inhaled glycerol aerosols generated by an e‐cigarette on the liver itself as well as circulating glucose and glycerol homeostasis. We found that inhaling aerosolized glycerol can affect circulating glucose and glycerol levels, as well as liver triglyceride concentration. We also found that young female mice were more susceptible to these effects. This is the first study investigating the specific impact of glycerol contained in e‐cigarette liquids on the liver and aspects of energy homeostasis.

Free glycerol levels are largely maintained by triglyceride hydrolysis (lipolysis) in adipose tissue during fasting (Rotondo et al., 2017). In this study, we found that inhaled glycerol e‐cigarette aerosols likely enter the blood stream, leading to a transient elevation of circulating glycerol levels in females but not in males, at least not at this concentration of aerosols. However, increase in blood glycerol following gavage is very similar between females and males, as well as the associated elevation in glucose. This suggests that the buffering capacity of males and females may be similar when glycerol is adsorbed through the digestive system but that, when entering from the lungs, sex differences can be observed. Aquaporins (AQP) such as AQP9 in the liver or AQP7 in adipose tissue facilitate glycerol transport through cellular membranes (Kuriyama et al., 2002). Obese‐ and insulin‐resistant mice show increased *aqp7* and *aqp9* expression, in spite of hyperglycemia (Hirako et al., 2016; Kuriyama et al., 2002). The lung also expresses several AQPs: AQP1 in microvascular endothelia, AQP3 in large airways, AQP4 in large‐ and small‐airway epithelia, and AQP5 in type I alveolar epithelial cells (Verkman et al., 2000). While AQP1, AQP4, and AQP5 function as selective water channels, AQP3 has been shown in human skin to transport glycerol (Boury‐Jamot et al., 2006; Hara‐Chikuma & Verkman, 2005). Glycerol can also passively permeate across membranes (Orbach & Finkelstein, 1980; Yang & Hinner, 2015). This suggests that less inhaled glycerol is “retained” by the lungs of female mice compared to male mice, leading to increased glycerol leakage to the blood stream in females. Additional research is required to better understand the fate of inhaled glycerol in the lungs.

In this study, we show that mice exposed to glycerol aerosols generated by an e‐cigarette do not gain nor lose significant weight, with no changes in adipose tissue weight or distribution. Glycerol uptake is regulated by insulin and is suppressed in fed state (Hibuse et al., 2006; Kishida et al., 2001; Rojek et al., 2007). In fasting state, glycerol is a key substrate for glucose production through the gluconeogenesis pathway (Baba et al., 1995; Landau et al., 1996). In post‐absorptive state, glycerol is used for lipid synthesis, such as triglycerides and phospholipids (Rotondo et al., 2017; Vance, 2015). High‐fat diets induce weight gain, mainly attributed to increased adipose tissue weight (Sundaram & Yan, 2016; Tan et al., 2017). Given that weights remained similar between exposed and control mice and that adipose tissues remained similar between groups, it does not appear that exposure to glycerol vaping would impact fat mass. However, further studies are needed to assess if early glycerol aerosol exposure can change food intake and energy expenditure.

High‐fat diet promotes nonalcoholic fatty liver disease (NAFLD), characterized by increased liver weight largely due to triglyceride accumulation (Asgharpour et al., 2016; Ishimoto et al., 2013; Tan et al., 2017). Insulin resistance is also associated with NAFLD, further increasing pathological fatty acid metabolism (Hwang et al., 2007). Interestingly, hepatic AQP9 protein levels, the main hepatic glycerol AQP, are inversely associated with the severity of hepatic steatosis, suggesting glycerol homeostasis affects and/or is affected by liver steatosis (Rodriguez et al., 2014). In this study, we found that exposure to glycerol e‐cigarette aerosols mildly affects glucose tolerance in young female and male mice. Moreover, we also found that female mice presented increased hepatic triglyceride and phosphatidylcholine levels despite no changes in liver weight, inflammation, remodeling, or endoplasmic reticulum stress. With unchanged fasting blood glucose, this hepatic lipid accumulation phenotype suggests that excess glycerol could be converted into lipids by the liver and stored there, representing an adaptive mechanism to maintain normal circulating glycerol levels. Interestingly, no increase in adipose tissue weight was observed, supporting that the adipose tissue is not the main site dealing with glycerol excess. The reason why this phenomenon is only observed in females remains unknown, however the amount of glycerol reaching the circulation from the lungs could provide some insight. While the liver phenotype observed in female mice does not appear to be pathogenic and males appear to be resistant, longer exposures over several months may be required to reach a pathogenic state in females and break the resistance in males. Moreover, investigating the impact of inhaled glycerol aerosolized by an e‐cigarette in mice developing and with an established NAFLD may show how vaping glycerol can affect prevalent liver diseases.

Circadian rhythm regulates a multitude of biological pathways, from our sleep and awake cycle, to our immune response to infection and to metabolic processes (Altman, 2016; Bass & Takahashi, 2010; Tan & Scott, 2014). In recent years, studies have associated the rise of metabolic syndrome prevalence with increased circadian rhythm deregulation due to light pollution, reduction in quality and quantity of sleep as well as jet lag due to work shifts or travel (Coomans et al., 2013). Among many other factors, it appears circadian rhythm plays an important role in the pathogenesis of metabolic syndrome (Garcia‐Rios et al., 2014). Our group reported that nicotine‐free and flavor‐free propylene glycol and/or glycerol aerosols induce changes in circadian rhythm regulatory gene expression in multiple organs in female BALB/c mice (Lechasseur et al., 2017, 2020). In this study, we also found that young but not older female and male C57BL/6 mice present hepatic changes in circadian gene expression levels. This suggests that the age of initiation has an impact on circadian changes observed, since the length of exposure for adult and young mice was the same. While it is possible that interactions between the circadian regulation of energy homeostasis and exposure to inhaled glycerol may be interacting more deeply in younger mice, our understanding of this interaction is currently very limited. Nevertheless, this finding is of great interest knowing how e‐cigarette use is highly prevalent in adolescents (Dai & Leventhal, 2019; Lin et al., 2020; Veliz et al., 2020).

Our group previously reported that an 8‐week exposure to flavor‐free and nicotine‐free propylene glycol and glycerol e‐cigarette aerosol increases larger airway resistance in female BALBC/c mice (Lechasseur et al., 2020). Others also evaluated the impact of flavor‐free and nicotine‐free e‐cigarette aerosols on lung functions (*reviewed in* Lechasseur and Morissette [2020]). However, previous studies did not assess specifically age and sex differences. In the present study, we found that lung functions from young males were more affected by exposure to glycerol emission of e‐cigarettes, with no changes observed in adult male mice or both female groups. This highlights that, along with our findings on the liver and energy homeostasis, age and sex differences are crucial variables in studying the effects of vaping, no matter to organ or the biological function studied. Also, the marked disconnection between the metabolic (females more affected) and the respiratory effects (young mice more affected) of glycerol‐based e‐cigarette emissions suggest pulmonary involvement does not mean systemic effects, and vice versa.

Some limitations can be identified in this study. We cannot know for certain that the increase in circulating glycerol levels following exposure to glycerol e‐cigarette aerosols represents the direct diffusion of inhaled glycerol from the airways to the blood. Use of labeled glycerol, such as C_13_‐glycerol, would allow determining this with certainty. Food intake was not measured in this study. Therefore, we do not know how much of what was observed can be attributable to changes in food intake in the e‐cigarette groups. Identifying the mechanisms behind our observations will require monitoring of food intake. Also, literature on the respiratory system as an entry point for glycerol is relatively inexistent and we could not address the mechanisms behind our observations. Further research will be required to identify the biological differences in inhaling versus ingestion glycerol.

This study shows that the glycerol contained in vaping liquids can affect the liver and aspects of energy homeostasis in a sex‐dependent manner without necessarily affecting lung functions. While additional studies are definitely needed to deepen our understanding or these observations, it shows how complex the biological impacts of vaping can be.

## CONFLICT OF INTEREST

The authors have no relevant disclosures or conflict of interest to declare.

## AUTHOR CONTRIBUTIONS

All authors contributed to the manuscript. Ariane Lechasseur, Mathieu Laplante, and Mathieu C. Morissette designed the study. Ariane Lechasseur, Mathilde Mouchiroud, Félix Tremblay, Gabrielle Bouffard, Nadia Milad, Marie Pineault, Michaël Maranda‐Robitaille, Joanie Routhier, Marie‐Josée Beaulieu, and Sophie Aubin performed the experiments. Ariane Lechasseur, Mathieu Laplante, and Mathieu C. Morissette analyzed the data. Ariane Lechasseur and Mathilde Mouchiroud drafted the manuscript. Mathieu Laplante edited the manuscript. All authors approved the final version of the manuscript.

## Supporting information



Fig S1

Fig S2
