# Supplementary material for: Glycerol contained in vaping liquids affects the liver and aspects of energy homeostasis in a sex‐dependent manner
Source: Physiol Rep. 2022 Jan 25;10(2):e15146. doi: 10.14814/phy2.15146 (PMC8787618; doi:10.14814/phy2.15146)
Supplement: Supplementary file 1 — Fig S1 [file PHY2-10-e15146-s002.pdf]

Figure S1

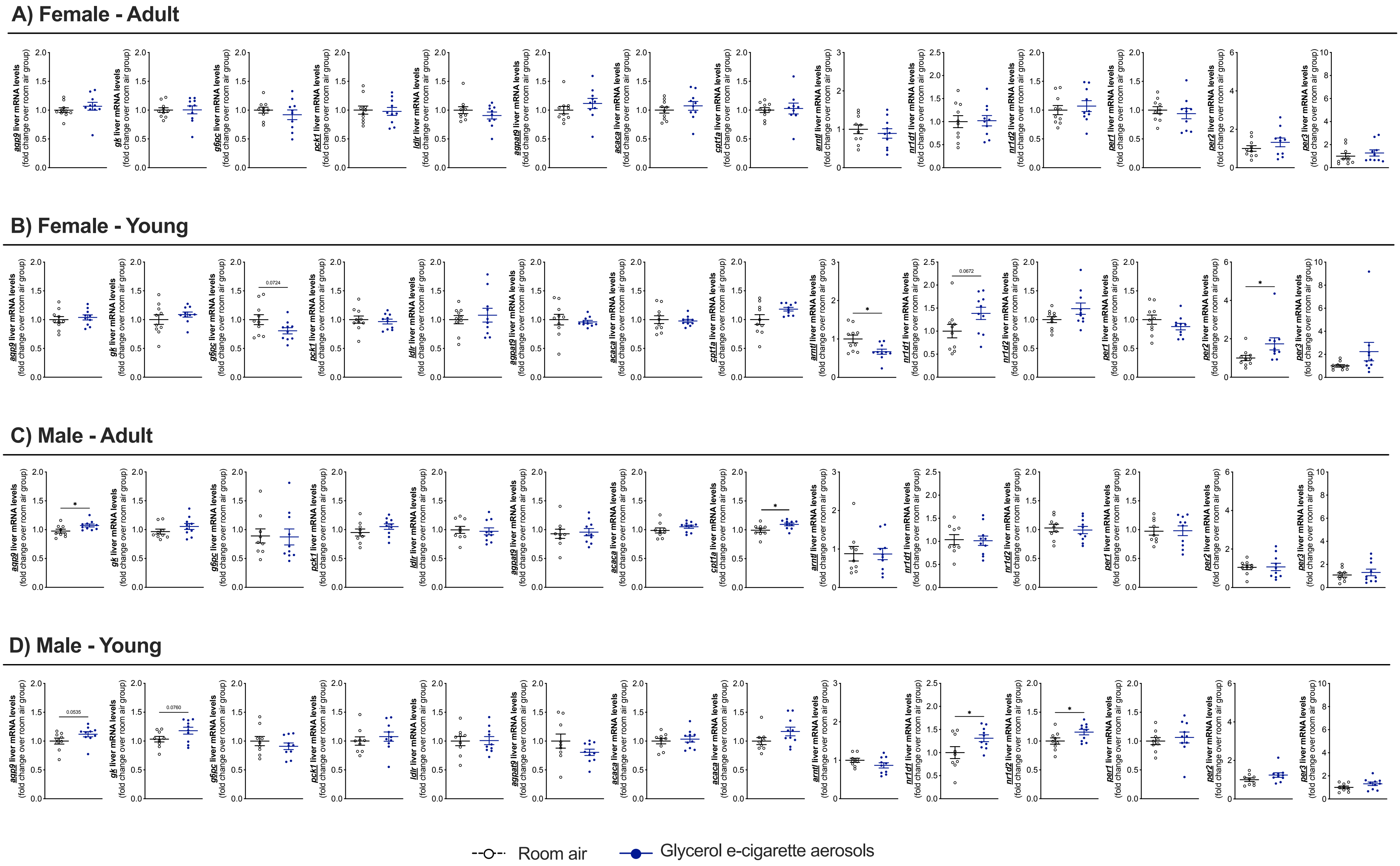

A) Female - Adult
B) Female - Young
C) Male - Adult
D) Male - Young
aqp9 liver mRNA levels (fold change over room air group)
gk liver mRNA levels (fold change over room air group)
g6pc liver mRNA levels (fold change over room air group)
pck1 liver mRNA levels (fold change over room air group)
ldlr liver mRNA levels (fold change over room air group)
agpat9 liver mRNA levels (fold change over room air group)
acaca liver mRNA levels (fold change over room air group)
cpt1a liver mRNA levels (fold change over room air group)
arntl liver mRNA levels (fold change over room air group)
nr1d1 liver mRNA levels (fold change over room air group)
nr1d2 liver mRNA levels (fold change over room air group)
per1 liver mRNA levels (fold change over room air group)
per2 liver mRNA levels (fold change over room air group)
per3 liver mRNA levels (fold change over room air group)
0.0724
0.0672
0.0535
0.0760
*
Room air
Glycerol e-cigarette aerosols
