# Supplementary figures and images for: Glycerol contained in vaping liquids affects the liver and aspects of energy homeostasis in a sex‐dependent manner
Source: Physiol Rep. 2022 Jan 25;10(2):e15146. doi: 10.14814/phy2.15146 (PMC8787618; doi:10.14814/phy2.15146)

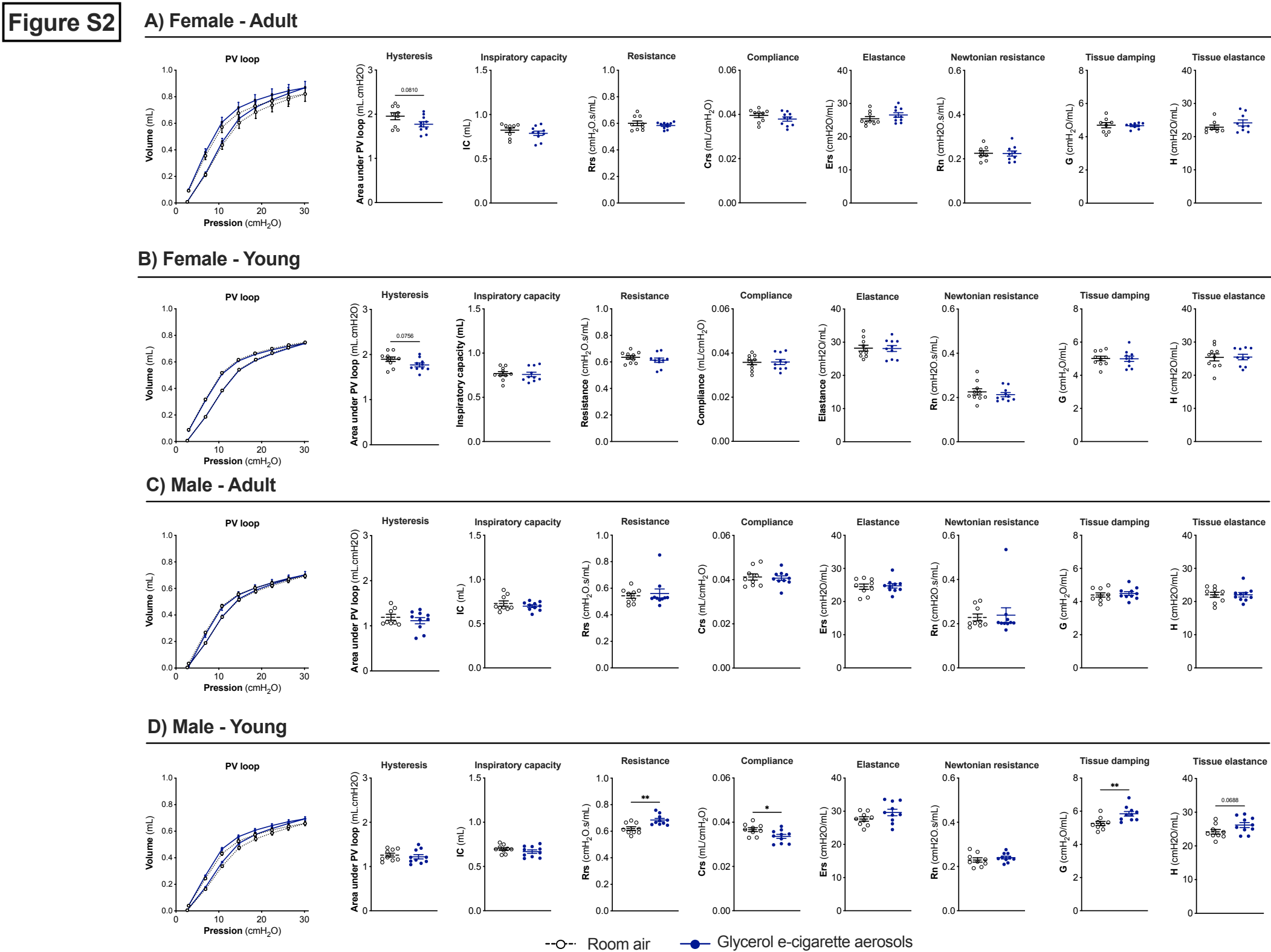

Supplement: Supplementary file 2 — Fig S2 [file PHY2-10-e15146-s001.pdf]
